# Novel Biocontrol Methods for *Listeria monocytogenes* Biofilms in Food Production Facilities

**DOI:** 10.3389/fmicb.2018.00605

**Published:** 2018-04-03

**Authors:** Jessica A. Gray, P. Scott Chandry, Mandeep Kaur, Chawalit Kocharunchitt, John P. Bowman, Edward M. Fox

**Affiliations:** ^1^CSIRO Agriculture and Food, Werribee, VIC, Australia; ^2^Centre for Food Safety and Innovation, Tasmanian Institute of Agriculture, University of Tasmania, Hobart, TAS, Australia

**Keywords:** *Listeria monocytogenes*, biofilms, biocontrol, bacteriophages, bacteriocins, endolysins, competitive exclusion, essential oils

## Abstract

High mortality and hospitalization rates have seen *Listeria monocytogenes* as a foodborne pathogen of public health importance for many years and of particular concern for high-risk population groups. Food manufactures face an ongoing challenge in preventing the entry of *L. monocytogenes* into food production environments (FPEs) due to its ubiquitous nature. In addition to this, the capacity of *L. monocytogenes* strains to colonize FPEs can lead to repeated identification of *L. monocytogenes* in FPE surveillance. The contamination of food products requiring product recall presents large economic burden to industry and is further exacerbated by damage to the brand. Poor equipment design, facility layout, and worn or damaged equipment can result in *Listeria* hotspots and biofilms where traditional cleaning and disinfecting procedures may be inadequate. Novel biocontrol methods may offer FPEs effective means to help improve control of *L. monocytogenes* and decrease cross contamination of food. Bacteriophages have been used as a medical treatment for many years for their ability to infect and lyse specific bacteria. Endolysins, the hydrolytic enzymes of bacteriophages responsible for breaking the cell wall of Gram-positive bacteria, are being explored as a biocontrol method for food preservation and in nanotechnology and medical applications. Antibacterial proteins known as bacteriocins have been used as alternatives to antibiotics for biopreservation and food product shelf life extension. Essential oils are natural antimicrobials formed by plants and have been used as food additives and preservatives for many years and more recently as a method to prevent food spoilage by microorganisms. Competitive exclusion occurs naturally among bacteria in the environment. However, intentionally selecting and applying bacteria to effect competitive exclusion of food borne pathogens has potential as a biocontrol application. This review discusses these novel biocontrol methods and their use in food safety and prevention of spoilage, and examines their potential to control *L. monocytogenes* within biofilms in food production facilities.

## Introduction

*Listeria monocytogenes* is a Gram-positive, rod shaped, facultative anaerobe capable of causing food borne illnesses particularly in high-risk population groups including the elderly, immune compromised, pregnant women, and neonates (Farber and Peterkin, 1991). While *L. monocytogenes* associated illness is not as common as that of other food borne pathogens like *Salmonella, Campylobacter*, or *Escherichia coli*, its mortality rate can be considered the highest. Approximately 30 % of invasive listeriosis cases lead to mortalities with most requiring hospitalization, and therefore demanding *L. monocytogenes* can be considered as a food borne pathogen of public health importance (Lomonaco et al., 2015; Véghová et al., 2016). Due to its ubiquitous nature, *L. monocytogenes* poses a food safety risk as it is frequently introduced into the processing environment through raw ingredients. *L. monocytogenes* can adhere to a variety of abiotic surfaces with some strains persisting for numerous years and acting as a source of continuous cross contamination (Fox E. et al., 2011; Coughlan et al., 2016; Colagiorgi et al., 2017).

Due to significant food safety risks, the control of *L. monocytogenes* has become a regulatory requirement that food business operators must adhere to. Regular cleaning, disinfecting, and sanitizing of food contact and non-food contact surfaces are required as part of a sanitation plan that also incorporates maintenance of equipment and buildings, pest control, and general hygiene. In addition, the implementation of good manufacturing practices and effective hazard analysis critical control point plan aids in reducing the risk of food borne illness (Drew and Clydesdale, 2015). However, *L. monocytogenes* is a difficult organism to eradicate and its presence still occurs even with the best management plans (Tompkin, 2002; Drew and Clydesdale, 2015).

While the exact mechanisms can be unclear for how *L. monocytogenes* is able to persist in food production environments (FPEs) so successfully, researchers have proposed that there are numerous factors at play. Poorly maintained equipment, surfaces, and unhygienic factory design can result in niches containing adequate nutrients, water, and protection from cleaning allowing bacteria to survive and grow while also introducing bacteria to subinhibitory levels of disinfectants (Carpentier and Cerf, 2011; Fox E.M. et al., 2011; Ibba et al., 2013; Møretrø et al., 2017). Typically disinfectants, when applied correctly, can sufficiently inhibit the colonization of introduced planktonic cells; however, dosing failures and applying disinfectants to wet surfaces can result in equipment being inadequately disinfected and bacteria being exposed to subinhibitory chemical levels (Martinez-Suarez et al., 2016; Møretrø et al., 2017). Incorporating desiccation processes has been shown to increase the effectiveness of disinfections procedures (Overney et al., 2017); however, an ample amount of drying time is difficult when continuous or even daily production runs are required. It is also important to note the difference between resistance, an increase in concentration or time required to exert the same reduction, and tolerance, an adaptation in a microbe’s susceptibility potentially the result of exposure to subinhibitory levels (Cerf et al., 2010; Ortega Morente et al., 2013). For example, some *L. monocytogenes* strains are known to carry genes for disinfectant chemical efflux pumps, such as *qacH* and *bcrABC*. The distribution of these genes tends to vary on a strain by strain basis instead of being unique to a specific lineage or subtype (Dutta et al., 2013; Ortiz et al., 2015; Møretrø et al., 2017). Although it has been reported that these genes only result in tolerance to quaternary ammonium compounds at levels far below the concentrations actually used in the food industry (Tezel and Pavlostathis, 2015), the ability to form biofilms is also a crucial factor in the survival of *L. monocytogenes.* Biofilms are composed of numerous cells attached to each other or an abiotic surface surrounded by an extracellular matrix containing a mixture of polysaccharides, proteins, and extracellular DNA (da Silva Fernandes et al., 2015; Fagerlund et al., 2017). This extracellular matrix provides a protective barrier around the internalized microbial cells from desiccation and heat, contributes to increased adhesion, and is a reservoir of nutrients (Colagiorgi et al., 2016). In addition, biofilms can impede the activity of antimicrobial agents as the matrix limits their diffusion potential and contains cells with differing susceptibility while also allowing for the acquisition of new genetic traits like those mentioned above through horizontal gene transfer. Further, biofilms typically consist of multiple species that can allow for the colonization of transient strains or provide increased attachment and survival to strains not typically good biofilm formers (Coughlan et al., 2016).

## The Biocontrol Methods Movement

While tolerance to disinfectants and sanitizers is not considered as significant an issue as antibiotic resistance, their continued use and potential ineffectiveness against biofilms warrant new strategies for the control of *L. monocytogenes*. As consumers become more conscious of food safety significance, the use of novel biocontrol methods is gaining further interest. This return to biocontrol methods of microbes and plants has the potential to relieve some of the tolerance to disinfectants and decrease some of the selective pressures that their overuse has on maintaining resistance markers (Coughlan et al., 2016). Biocontrol methods with potential to act against listerial biofilms include bacteriophages, their endolysins, competitive bacterial species and their antimicrobial products, bacteriocins, and plant-derived products and will be discussed in this review.

## Bacteriophages

**FIGURE 1:**
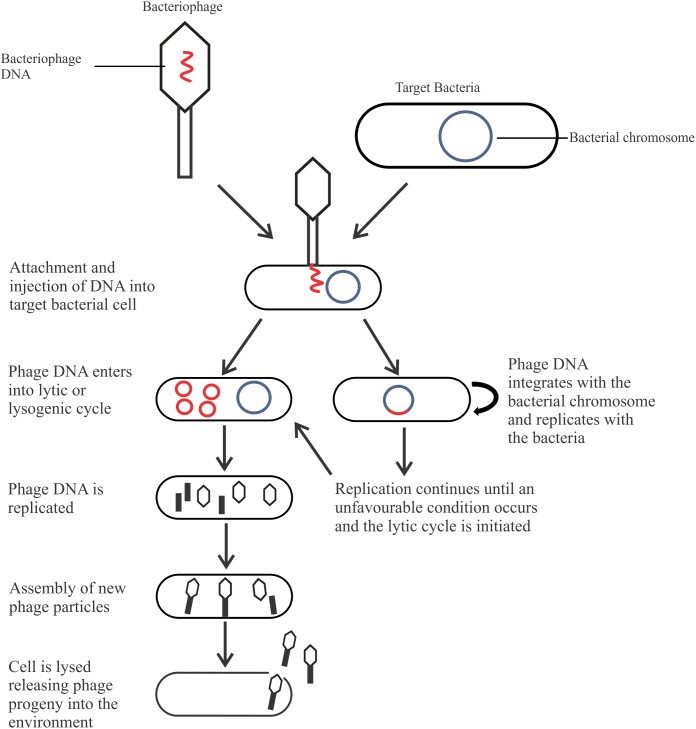
The life cycles of a bacteriophage.

The most abundant microorganism on earth, bacteriophages (phages) are viruses that infect bacteria for propagation, live naturally in the environment, and anywhere host bacteria are found (Bai et al., 2016; Pérez Pulido et al., 2016). Phages are classified based upon their morphology (head and tail, either contractile or non-contractile, or no tail), nucleic acid (double stranded or single stranded; deoxyribonucleic or ribonucleic acid), and life cycle, which is of most relevance for biocontrol. There are two types of life cycles phages can undergo after entering the bacterial cell: the lysogenic cycle (temperate phages) or the lytic cycle (**Figure 1**). Phages may be capable of a lysogenic cycle that converts to the lytic cycle in unfavorable conditions, or undergo a solely lytic life cycle. Temperate phages are not suitable as a biocontrol agent as integration into the host genome may result in increased pathogenicity through horizontal gene transfer (Hagens and Loessner, 2007; Salmond and Fineran, 2015). In comparison, lytic phages are ideal as a biocontrol agent due to their fast-lytic action.

Although identified over a hundred years ago, interest in phages has only recently been reignited with the rise of antibiotic resistance among bacteria (Hagens and Loessner, 2007). The utility of phages has included the treatment of diseases in humans and animals, typing of bacterial strains, decontaminating meat carcasses after slaughter, and targeted inactivation of pathogenic and spoilage bacteria on food contact and non-contact surfaces as well as surfaces of ready to eat products and during packaging and storage (Hagens and Loessner, 2007; Strauch et al., 2007). The application of phages as an innovative approach to control biofilms in the FPE is also beginning to be explored. While there has been great achievement in the use of phages from a therapeutic perspective, their success in the FPE is not as simple. Factors like the composition and structure of the biofilm, temperature, the metabolic state of the bacteria in the biofilm, the extracellular matrix in general, food components, and nutrients all provide additional challenges to the effectiveness of phage application (Parasion et al., 2014). While there have been some reports of phage resistance (Fister et al., 2016), it occurs more gradually than the development of antibiotic resistance as phages are able to mutate continuously, like bacteria, and resistance is further slowed by using a combination of phages active against the one bacterial species (Hagens and Loessner, 2007; Sadekuzzaman et al., 2017). There is a substantial amount of research conducted on phages’ ability to protect food from *Listeria*, with two commercial *Listeria* phage products, ListShield^TM^ and Listex^TM^ P100 approved as food preservatives with the generally recognized as safe status since 2006. However, studies investigating the efficacy of these products and other *Listeria* phages against biofilms are few, with most having focused on Listex^TM^ P100.

Biofilm maturity has the potential to reduce the efficacy of phage treatment, as well as any control method. Various studies have examined this concept utilizing preformed biofilms at various maturity levels, ranging from 24 h to 2 weeks, with most studies reporting a minimum 1-log reduction. Most studies to date have utilized stainless steel as the surface to form *L. monocytogenes* biofilms and examine the efficacy of bacteriophage treatments. This reflects the widespread presence of these surfaces, both food contact and non-contact in food processing environments. The success of bacteriophage treatments at inactivating *L. monocytogenes* biofilms on these surfaces, however, has shown mixed results. A number of studies demonstrated promising results when Listex^TM^ P100 was applied to *L. monocytogenes* biofilms on stainless steel, with reductions in the order of 5-log being achieved (Soni and Nannapaneni, 2010; Montañez-Izquierdo et al., 2012). Both of these studies used an application treatment of 24 h at ambient room temperature. Iacumin et al. (2016) also applied Listex^TM^ P100 for 24 h at 20°C onto stainless steel wafers and report the complete elimination of *L. monocytogenes* biofilm. This prolonged treatment application, however, in many cases is not practical in an FPE. In addition, Iacumin et al. (2016) pressed the stainless steel wafer onto an agar plate to replicate the process of cross-contamination in the FPE; however, it did not take into consideration the phage products ability or inability to act on biofilms in the crevices or corners where these might be thicker than a flat surface.

A shorter treatment time of 2 h was applied by Sadekuzzaman et al. (2017) when running a similar inactivation test with ListShield^TM^; however, this was associated with a much lower inactivation of just 2-log when applied to *L. monocytogenes* biofilm on stainless steel. This was even less effective on a rubber surface, achieving a 1-log reduction in *L. monocytogenes* cell numbers. The results of Sadekuzzaman et al. (2017) also reflect those observed by Gutiérrez et al. (2017) who saw a similarly low inactivation achieved by a 4 h ListShield^TM^ treatment, typically 1-log or less. Although the latter study did show greater inactivation with Listex^TM^ P100 under the same treatment conditions, the Listex^TM^ P100 commercial phage preparation showed a reduced activity range, only capable of infecting 7 of the 11 strains tested. An important aspect in phage application is the ratio of phage to bacteria known as the multiplicity of infectivity. To increase the likelihood the phage will infect the bacterium, the phage needs to be at a higher ratio than the number of target bacterial cells (Montañez-Izquierdo et al., 2012). High multiplicity of infectivity has been reported to result in efficient phage treatment with one study recommending a multiplicity of infectivity around five was required for adequate reductions by Listex^TM^ P100 (Montañez-Izquierdo et al., 2012).

Apart from temperature, multiplicity of infectivity, and treatment time, other factors may influence efficacy of biocontrol treatments, notably the presence of organic matter such as the food matrix. A further parameter which must be considered when examining efficacy of treatment on surfaces is the surface architecture itself, which may range from a smooth rendered surface to a scored surface with associated crevices which may be colonized by bacteria and their biofilms. Chaitiemwong et al. (2014) considered both surface crevices and food matrices (diluted food residues of ham, salmon, endive, or milk) when measuring the efficacy of Listex^TM^ P100 treatment. Results suggested deeper crevice features on the surface decreased the treatment efficacy, with inactivation in the magnitude of > 3-log achieved on 0.2 mm crevices compared to the max 1.4-log CFU/mL observed in crevice depth of 5 mm. Of particular note was the difference seen when comparing the food matrix, with lower inactivation observed for milk and vegetable when compared with meat or fish. Ganegama Arachchi et al. (2013) mimicked conditions in fish processing and demonstrated the presence of fish protein led to a lower associated biofilm density compared to control stainless steel experiments when a fish protein matrix was added to the cultivation of *L. monocytogenes* biofilm on stainless steel. This highlights the complex role the food matrix may play in both biofilm formation and subsequent efficacy of bacteriophage treatment, demonstrating the need for further studies to understand the significance of food matrix on bacteriophage treatment efficacy.

Taken together, current literature detailing phage biocontrol studies directed at *L. monocytogenes*, such as those detailed above, shows differing success in their ability to decrease established biofilms. The often low reductions achieved demonstrate the challenges biofilms pose for not only bacteriophages but all control methods, but this is not to say that there is no place for phages as a potential biocontrol method. As with many disinfection regimes, additional interventions such as steps to loosen biofilm or remove organic matter can increase the success of phage treatments (Ganegama Arachchi et al., 2013). Further research considering multi-species biofilms and in-facility application will help determine the true potential of this biocontrol approach.

## Endolysins

Endolysins (lysins) are hydrolytic enzymes required for bacteriophage dissemination from the host bacterial cell. They occur at the end of the lytic cycle to release the phage virions by breaking down peptidoglycan in the bacterial cell wall in what is termed lysis from within (Chan and Abedon, 2015; Schmelcher and Loessner, 2016). Researchers have harnessed lysins through protein expression production systems, generally in *E. coli*. Following purification of the lysin, it can be applied externally to the cell wall, thus not requiring phage infection, for biopreservation and biocontrol application (García et al., 2010). Lysins are grouped based upon the cell wall component they attack with the five main classes being N-acetylglucosaminidases, endo-β-N-acetlyglucosaminidases, lytic transglycosylases, endopeptidases, and N-acetylmuramoyl-L-alanine amidases (García et al., 2010; Schmelcher and Loessner, 2016). Lysins are highly specific with a narrow spectrum of activity making them host specific with some lysins only being active on the bacterial strain the phage was isolated from (Oliveira et al., 2012). In addition, they are fast acting and no development of resistance has been reported to date (Schmelcher and Loessner, 2016). Most research has occurred on Gram-positive bacteria using the lysis from without approach as the peptidoglycan layer is exposed. Although limited, antimicrobial activity of lysins on Gram-negative bacteria has been reported when used in conjunction with EDTA, a membrane permeablizer (Chan and Abedon, 2015).

The antimicrobial activity of lysins has mostly focused on infection control of staphylococcal bacteria. Other applications that have been considered include use in agriculture to prevent plant disease by either intense application of cell lysates expressing a chosen lysin or development of transgenic plants by incorporation of the lysin gene into the plant genome (Düring et al., 1993; Kim et al., 2004); as a rapid detection and imaging method of pathogenic bacteria (Schmelcher et al., 2010; Bai et al., 2016); and transformation of listerial bacteriophage endolysin encoding genes into dairy starter cultures as a biopreservation method (Gaeng et al., 2000). Antilisterial lysins isolated to date have predominately focused on the control of planktonic cells *in vitro* with promising results although further validation is required (**Table 1**). Only a few antilisterial lysins have been assessed in food products and the food matrix and environment have been found to affect the antimicrobial activity (Oliveira et al., 2012).

**Table 1:** Antilisterial lysins reported in literature, key summary, and application.

Endolysin	Reported findings	Use	Reference
Ply118	Rapidly lysed all Listeria strains tested and against three Bacillus species.	BC, IC	Loessner et al., 1995
Ply500		BC, IC	
Ply511	Rapidly lysed all Listeria strains tested against.	BC, IC	
PlyP35	Determined optimal temperature, NaCl, pH, and various ions conditions.	BC, IC	Schmelcher et al., 2012
PlyP40	Lysed *L. monocytogenes* strain and *L. innocua*.	BC, IC	Loessner and Schmelcher, 2010
PlyP825	Inhibited all growth in *L. monocytogenes* strains used.	BC, IC	Grallert et al., 2012
PlyPSA	Determined crystalized structure	RMD	Korndörfer et al., 2006
PlyP100	Lysed all *L. monocytogenes*, Listeria strains in cheese, and a *Bacillus subtilus* strain tested against	BC, BP	Van Tassell et al., 2017
LysZ5	Lysed *L. monocytogenes*, *L. innocua*, and *Listeria welshimeri*; reduced *L. monocytogenes* numbers in soya milk.	BC, BP	Zhang et al., 2012
PlyLM	Lysed all *L. monocytogenes* and *L. innocua* strains tested against; digested *L. monocytogenes* biofilms when combine with a protease.	BC	Simmons et al., 2012

*BC, biocontrol; IC, infection control; RMD, rapid multiplex detection; BP, biopreservation*.

To date there is only one lysin, PlyLM, which has been tested against *L. monocytogenes* biofilms after 100 % susceptibility on planktonic *L. monocytogenes* and *Listeria innocua* cells was achieved (Simmons et al., 2012). PlyLM reduced the monolayer biofilm to the same level as the application of lysozyme and proteinase K. When used in combination with proteinase K, or both proteinase K and lysozyme, synergistic effects were observed, and the biofilm was effectively digested. However, biofilms were only grown for 24 h at 37°C, and therefore the efficacy of these enzymes under other conditions merits further investigation, for example, performance at lower temperatures which are more reflective of those of most FPEs. More research has been undertaken on staphylococcal biofilms, predominantly monospecies biofilms, which have achieved reductions in biofilm mass. Of interest is their efficacy against persister cells. Persister cells are metabolically inactive subpopulations of cells, which are “super-resistant” to antimicrobial agents such as antibiotics (Brooun et al., 2000; Wood, 2017). Studies have shown these persister cells occur as a subpopulation of bacterial biofilms, and as such can present a significant obstacle to biofilm inactivation by antimicrobials (Brooun et al., 2000; Singh et al., 2009). Several studies have shown a promising role for lysins to inactivate persister cells in biofilms (Gutiérrez et al., 2014; Schuch et al., 2017). The success being reported against staphylococcal biofilms suggests that the potential lysins may have against biofilms in a food production context, particularly in targeting *Listeria* biofilms, which are a significant problem in FPEs. Another phage enzyme, extracellular polysaccharide depolymerase, has also be shown to degrade biofilm EPS; however, they are highly specific to the strains the phage infects (Chan and Abedon, 2015). A similar approach targeting *L. monocytogenes* in biofilms could also present an alternative control measure.

## Competitive Bacterial Species

Competitive exclusion is where one bacterial species competes with another species over resources and/or space in a habitat, successfully reducing the number of cells or excluding that species (Hibbing et al., 2010). This competitive exclusion can be the result of the production of antimicrobials such as bacteriocins, organic acids either acting directly against the species it is competing with or acting on the environment altering the pH, or alternatively physically outcompeting other bacterial species for nutrients and/or space and limiting normal survival or proliferation of those competitive species. This strategy is typically categorized into three components: competition, where planktonic cells of both species are co-cultured for a period of time; exclusion, where the antagonistic species are grown to a biofilm cell density prior to the addition of planktonic cells of the target species; or displacement, in which the target species are grown to biofilm cell density prior to addition of planktonic antagonists (Woo and Ahn, 2013; Pérez-Ibarreche et al., 2016). As biofilms protect microorganisms from chemical cleaners and disinfectants, the use of non-pathogenic microorganisms may assist sanitation approaches in controlling, preventing, or eradicating unwanted species like food borne pathogens.

Competitive exclusion studies typically pit planktonic cells of the antagonist species (i.e., the species which will exert a competitive exclusion effect) against planktonic cells of the target species in a competition assay, grown together for a period of time facilitating biofilm formation. Daneshvar Alavi and Truelstrup Hansen (2013) used a short incubation time of 72 h which resulted in a 1-log decrease in *L. monocytogenes* cell density after application of *Serratia proteamaculans.* A similar reduction was also reported by Fox et al. (2014) of *L. monocytogenes* biofilm cell density after 96 h when grown in co-culture with *Janthinobacterium lividum*. However, greater reductions have been reported when cells were incubated for longer periods with results around log 4.5 and 5.5 on stainless steel coupons and polytetrafluoroethylene, respectively (Pérez-Ibarreche et al., 2016). Zhao et al. (2004) also reported higher magnitude reductions of 7.8-log reduction over 28 days at 15°C by two bacterial isolates, *Lactococcus lactis* (*Lc. lactis*) and *Enterococcus durans*. In another experiment performed at 8°C for 28 days, four isolates, including the previous two isolates were also capable of reductions around 7-log units. However, the higher reductions reported by Zhao et al. (2004) and Pérez-Ibarreche et al. (2016) were produced by lactic acid bacteria (LAB) whose inhibitory activity has been studied extensively for many years, particularly as probiotics (Jeong and Frank, 1994).

The inhibitory effect of LAB was further explored by Guerrieri et al. (2009) and Gómez et al. (2016) as a preformed biofilm preventing *L. monocytogenes* biofilm formation as part of the exclusion strategy. Gómez et al. (2016) tested a variety of LAB strains and found reductions ranged from 4- to 7-log units over 24 and 48 h; however, by 72 h, *L. monocytogenes* growth had increased by almost half fold of the control indicating that these strains were only capable of exclusion within the first 24–48 h. However, *Lc. lactis* 368 strain was able to completely exclude the growth of *L. monocytogenes* for the entire period, although it should be noted that all experiments were performed at a relatively elevated temperature and as such lower temperatures reflective of many FPEs require further consideration. In comparison, Guerrieri et al. (2009) showed the potential of LAB bacteria at refrigeration temperatures with a *Lactobacillus plantarum* (*Lb. plantarum)* strain capable of a 4-log reduction over a 10-day period. Mariani et al. (2011) used the native biofilm microflora of wooden cheese ripening shelves to achieve a 1- to 2-log reduction over a 12-day period, although this reduction was less than that observed in Guerrieri et al. (2009) and Gómez et al. (2016).

The third strategy displacement, as reviewed by Woo and Ahn (2013), demonstrated that the use of planktonic antagonist LAB strains as a post-treatment control method targeting *L. monocytogenes* was less effective compared to pre-treatment, although two strains (*Lactobacillus paracasei* and *Lactobacillus rhamnosus*) were capable of a 3-log reduction in *L. monocytogenes* biofilm cell density over 24 h when incubated at 37°C.

While most studies are performed in laboratories, Zhao et al. (2006, 2013) took the concept of competitive exclusion a step further and looked at its applicability in poultry processing facilities. In a fresh poultry facility, two LAB strains (*Lc. lactis* and *E. durans*) were added to two enzyme-based cleaners and applied as a foam to selected drains four times in the first week and then two times for the following 3 weeks. Sampling continued for 18 weeks after the last treatment. Most drains experienced significant reductions within the first week after only four applications and all drains maintained lower levels of *Listeria* throughout the sampling period (Zhao et al., 2006). Importantly, two drains reported significant reductions 16 weeks after treatments finished. Similar parameters were applied to the application of the same strains at a ready to eat poultry processing facility. By the end of the first week of application, *Listeria* was not detected in five of the six drains with all drains reporting negative results between weeks 8 and 13 (Zhao et al., 2013). It should also be noted that the strains utilized were known to either possess nisin or other forms of antimicrobials; however, it was not elucidated if the inhibition was the result of the production of antimicrobials.

There have been some encouraging results in the use of LAB against *L. monocytogenes* biofilm cells in laboratory-based experiments (**Table 2**); however, very few have been trialed in actual FPEs, apart from Zhao et al. (2006, 2013). The results from their two studies have shown promising results as an alternative control method utilizing *E. durans* and *Lc. lactis*; however, further longitudinal research surrounding the in-facility application is required. In addition, the application of other bacterial species identified in some of the studies mentioned above, for example, *J. lividum* and *S. proteamaculans*, warrants in-facility testing. However, it should be noted that the LAB strains utilized for in-facility application studies were isolated from the production environment indicating that specific strains may work best in the environment they were isolated from and these strains may vary depending on the food industry.

**Table 2:** Bacterial species active against *L. monocytogenes* and purported mode of action.

Bacterial species	Mode of action	Studies
*S. proteamaculans*	Sánchez et al. (2010) identified a bacteriocin-like substance was produced at low temperatures capable of inhibiting *L. monocytogenes*. Inhibition was suggested to be the result of Jameson effect.	Sánchez et al., 2010; Daneshvar Alavi and Truelstrup Hansen, 2013
*J. lividum*	Specific strain utilized not tested for antimicrobial compounds. *J. lividium* are reported to have antibacterial compounds capable of inhibiting Gram-positive bacteria (O’Sullivan et al., 1990).	Fox et al., 2014
*Lc. lactis*	Neither of the studies by Zhao et al. tested for production of a bacteriocin; however, this species has previously be reported to produce nisin.	Zhao et al., 2004, 2006, 2013
*E. durans*	Neither of the studies tested for the bacteriocin; however, this species has previously be reported to produce enterocin.	Zhao et al., 2004, 2006, 2013
*Lb. plantarum 396/1*	Inhibition was attributed to production of an organic acid.	Guerrieri et al., 2009
*Lb. paracasei*	May be the result of competition for sites and resources. As a probiotic strain it may produce bacteriocin, organic acid or hydrogen peroxide.	Woo and Ahn, 2013
*Lb. rhamnosus*	May be the result of competition for sites and resources. As a probiotic strain, it may produce bacteriocin, organic acid or hydrogen peroxide. A previous study isolated an antilisterial bacteriocin from this species (Jeong and Moon, 2015).	Woo and Ahn, 2013
*Lb. sakei*	Bacteriocin producing strain.	Pérez-Ibarreche et al., 2016
*LAB – Lc. lactis 368*, *Lb. helveticus 354*, *Lb. casei 40*, and *W. viridescens 113*	Not identified as bacteriocin-producing strains. May be result of biosurfactants, or exclusion by trapping (killing cells embedded in biofilm).	Gómez et al., 2016
Native microbial flora of cheese ripening wooden shelves	Established biofilms on active cheese ripening wooden shelves were used. Inhibition may have been the result of competition for sites and nutrients.	Mariani et al., 2011

Houry et al. (2012) reported the use of bacterial species in a novel biocontrol approach. In the study, they identified a subpopulation of bacilli known as bacterial swimmers which were capable of creating transient pores within the biofilm structure. By pre-treating *Staphylococcus aureus* biofilms with bacterial swimmers, which also produced an anti-stapylococcal bactericide, they achieved a greater inactivation of *S. aureus* in biofilm by facilitating access of toxic substances in the environment into the biofilm.

## Bacteriocins

An important component of the competitive survival strategy of bacteria is the production of antimicrobial products. One group of ribosomally synthesized antimicrobials are the heat stable peptides known as bacteriocins (Cotter et al., 2005; Gálvez et al., 2008; Winkelströter et al., 2015). It has been suggested that most bacteria produce at least one bacteriocin and LAB are known to be prolific producers (Cotter et al., 2005). Most bacteriocins have a narrow spectrum of activity, that is, they are active against the same species that produces them but the producer is immune to them, while some have a broad spectrum of activity acting on members within the same genus as well as other genera and species (Cotter et al., 2005). The mode of activity varies depending on the particular class of bacteriocin and can include pore formation, or inhibition of key cellular processes such as peptidoglycan production, DNA replication, mRNA, or protein synthesis, to name a few (Cotter et al., 2005). There are two main groups: Class I (also known as lantibiotics), peptides that undergo post-translational changes, and Class II, which do not (Cotter et al., 2013). Among the most well-characterized and successful bacteriocins to date is nisin, a Class I bacteriocin from *Lc. lactis* which has been approved for use in food as a preservative/additive by the World Health Organization, European Union, and the United States Food and Drug Authority (Cotter et al., 2005). A great deal of research has gone into identifying more bacteriocins active against *L. monocytogenes* planktonic cells and biofilms, an important arena as nisin resistance is slowly being reported.

Most studies can be classified into two groups based upon how the bacteriocin is applied: either as whole bacterial cells known or suspected of bacteriocin production, or alternatively the bacteriocin extract itself, applied either as a crude or semi-purified product. Their utility against preformed *L. monocytogenes* biofilms of varying times has been the subject of numerous studies, with some reporting promising results. For example, Gómez et al. (2016) assessed *Lc. lactis*, *Lactobacillus sakei*, and *Lactobacillus curvatus*, all known to produce nisin Z, sakacine A, and sakacine P, respectively, against 48 h preformed biofilms. *Lb. sakei* and *Lb. curvatus* were capable of complete inactivation over 72 h whereas the two *Lc. lactis* strains provided a 6-log reduction by the end of the test period. Winkelströter et al. (2015), however, were unable to produce results of a similar magnitude when *L. monocytogenes* was co-cultured with *Lb. paraplantarum*, only achieving 2-log inactivation at 24 and 48 h before decreasing by 72 h. Guerrieri et al. (2009) took an alternative approach and reported that *Lb. plantarum* and *Enterococcus casseliflavus* were able to inactivate *L. monocytogenes* 7-day preformed biofilms by 3.9- and 3.7-logs over a 10 day-period. Importantly, the results could be associated with bacteriocin production, as no changes to the pH were observed.

Another technique is extracting the bacteriocin in the form of cell-free supernatant (CFS), as a crude bacteriocin fermentate or semi-purifying the product. The antimicrobial activity of CFS has shown mixed success in co-inoculation studies to prevent the formation of biofilms by *L. monocytogenes*, with Camargo et al. (2016) reporting significant reductions after 24 h, whereas Bolocan et al. (2017) only observed between 1.6- and 3.6-log CFU/cm^2^ reduction after 72 h depending on the media used. In the latter study, however, the CFS extract which produced the highest reduction was from an isolate known to also produce an organic acid which was not removed, and therefore this result may not be associated solely to the antimicrobial activity of the bacteriocin. When Camargo et al. (2016) applied the CFS to 24 h preformed biofilms for 2.5 h, they found biofilm formation continued in some isolates.

Other researchers have compared the two methods, bacterial cells and extracts again with varying results. García-Almendárez et al.’s (2008) analysis on 4-day preformed biofilms demonstrated a crude bacteriocin fermentate from *Lc. lactis* known to produce nisin A was capable of a 2.7-log reduction over 24 h. However, a greater reduction over 5-logs was achieved when the *Lc. lactis* was applied for 6 h, then rinsed, and placed in a desiccator for five days. Whereas, Winkelströter et al. (2011) co-inoculated *L. monocytogenes* with *Lb. sakei* or its CFS and found that any decreases observed in the first 24 h were diminished with time, as results at 48 h were comparable to the pure culture levels. A promising approach by Pérez-Ibarreche et al. (2016) involved the supplementation of *Lb. sakei* cells with a semi-purified bacteriocin for 6 h, which resulted in a twofold reduction in *L. monocytogenes* numbers on the stainless steel surface, or an additional 1-log reduction on polytetrafluoroethylene.

As mentioned previously, the bacteriocin nisin has been approved for commercial purposes and has paved the way as an alternative biocontrol method. Research into bacteriocins has been performed with comparable results to the other non-commercial bacteriocins discussed above. Minei et al. (2008) found that nisin was capable of inhibiting *L. monocytogenes* biofilm formation for 9 h on stainless steel coupons, and although cell growth did recommence after this time, a 3.5-log inactivation was still maintained by 48 h. On the other hand, Henriques and Fraqueza (2017) shortened the treatment time to 5 min and even at the highest concentration, no activity was recorded, although activity was defined as a ≥ 5-log decrease.

From the above, it is obvious that results vary significantly depending on if bacteriocin producing bacterial cells or the bacteriocin extracts is used. Results from bacteriocin extracts can be correlated to the antimicrobial action of the bacteriocin with greater certainty; however, additional analysis is required particularly when whole cells are used to help ensure that the measured inhibition is not the result of competitive exclusion or the production of other antimicrobials such as organic acids. The co-inoculation and preformed biofilm studies reflect the ability of the bacteriocin to either prevent the formation or affect the removal of established biofilms in the FPE; however, the length of time the biofilms are grown for prior to the bacteriocin being applied also affects the antimicrobial activity as mature biofilms may provide better resistance. Although several studies show that promising results most require additional analysis at temperatures and other environmental conditions mirroring the FPE to identify potential candidates suitable for further testing. With the potential resistance to nisin arising, the identification of other bacteriocins is essential. In addition, the application of synergistic antimicrobials to further combat the development of resistance should be considered.

## Plant-Derived Antimicrobial Products – Essential Oils

An alternative to the use of chemicals, microorganisms, or their derivatives is the use of plant-derived antimicrobial products such as essential oils (EOs). Herbs and spices are commonly known to exhibit antimicrobial activity and have been used by various cultures for flavoring, as a food preservative or for medicinal purposes. EOs play a key role in protecting plants from bacteria, fungi, viruses, insects, and animals (Perricone et al., 2015). Traditional distillation, cold press/expressing, solvent extractions, and enfleurage methods have been used to extract EOs from plant-derived materials; more recently, modern techniques including microwave or ultra sound assisted extraction, pressurized extractions, and super critical fluid extraction have been used to obtain EOs from a variety of plant sources (including roots, wood, bark, twigs, leaves, seeds, buds, flowers, and fruits). However, the constituents and compositions of EOs vary significantly from high concentrations to trace amounts based upon the plant part, plant age, and extraction method used, in turn influencing their antimicrobial activity (Lemberkovics et al., 2004; Reyes-Jurado et al., 2014; Perricone et al., 2015; Xia et al., 2017). Key molecules in EOs with the most effective antibacterial activity are typically from aldehyde and phenol chemical classes which include compounds such as cinnamaldehyde, carvacrol, eugenol, or thymol (Bakkali et al., 2008; Perricone et al., 2015). EOs are able to permeabilize the cell membrane resulting in the leakage of ions or other cell content, and may also disrupt key genetic functions and/or cellular components like proteins, polysaccharides, phospholipids, fatty acids, and essential enzymes due to the lipophilic nature of EOs (Bakkali et al., 2008; de Oliveira et al., 2010, 2012a; Perricone et al., 2015).

While there are thousands of EOs described, it is reported around 300 of these have generally recognized as safe approval and are used commercially for flavoring or fragrance; however, more detailed information is required for their use as a biocontrol agent (Burt, 2004; Reyes-Jurado et al., 2014). Most research surrounding the antimicrobial activity of EOs focuses on their effects on planktonic cells of food spoilage and pathogenic bacteria either in standard laboratory conditions or in their application on food items. This application on food as a biocide has major limitations as higher concentrations are required potentially interfering with the sensory attributes of the food (Burt, 2004; Chorianopoulos et al., 2008). In addition, some components of food items, mainly fats, proteins, carbohydrates, water, salt, antioxidants, pH, and other preservatives or additives used may impact upon the activity of the EOs (Perricone et al., 2015). Further research is required to understand the impact EOs have on bacterial pathogens and in particular their ability to prevent or eradicate biofilms in FPEs. Some research is occurring within this space; however, there is limited research against *L. monocytogenes* biofilms with a few studies looking at the extracted EOs, the active components of specific EOs, or altering the EO chemical composition. de Oliveira et al. (2010) assessed the EOs from fresh citronella (*Cymbopogon nardus*) and lemongrass (*Cymbopogon citratus*) leaves applied alone or in combination; however, it was the Citronella EO which demonstrated the highest reductions against both the 3 and 240 h preformed biofilms with complete reduction after 60 min of application. Similar results reported in another study by de Oliveira et al. (2012b) found 2% (vol/vol) Chinese cinnamon extract (*Cinnamomum cassia*) was capable of reducing a 48 h preformed biofilm to below the detection limit (2.84-log CFU/cm^2^) after 20 min; however, both of these studies applied the EOs at temperatures above 20°C.

Essential oils contain a mixture of major and minor molecules responsible for their antimicrobial activity with some of the major components being explored further. The active components of clove (eugenol) and spearmint (carvone) EOs were tested on a 6 h preformed *L. monocytogenes* biofilm but were found to increase biofilm mass by Leonard et al. (2010). Citral and nerol, in contrast, both major components from lemongrass (*C. citratus*) and *Lippia rehmannii* (nerol only), displayed a similar reduction as the positive control ciprofloxacin.

Additional microbial species can also impact upon the activity of the EO or active component. For example, Leonard et al.’s (2010) study as mentioned above was on *L. monocytogenes* monospecies biofilms and reported a mixture of results among the EO and the various active components tested, whereas de Oliveira et al. (2012a) looked at the activity of Chinese cinnamon and its active component, Cinnamaldehyde, on a mixed biofilm of *L. monocytogenes* and enteropathogenic *E. coli* on stainless steel coupons dipped in reconstituted whole milk. The EO and cinnamaldehyde were both capable of reducing the mixed biofilm to below the detection limit of 2.84-log CFU/cm^2^ whereas the EO and active components only provided reductions just over 2-logs on the *L. monocytogenes* biofilm. Chorianopoulos et al. (2008) examined the EO and hydrosol (by-product of the steam distillation) of *Satureja thymbra* (Pink Savory) and showed similar results when grown in a mixed biofilm with a food borne pathogen (*L. monocytogenes* and *Salmonella enterica*) and a spoilage bacterium (*Pseudomonas putida*). It was noted that the optimized application time was 60 min and any increase in time provided no additional reduction. The impact other microbial players may have on the activity of EOs requires further exploration in order to gain insights into the various relationships at play.

A common problem for the use of EOs as a biocontrol method on food products is the associated impacts on taste at concentrations required for appropriate antimicrobial effect. A process to concentrate the EOs for application at a lower volume with the same potentially high antimicrobial activity may be required in the case of some EOs. Krogsgård Nielsen et al. (2017) looked at emulsifying and encapsulating isoeugenol oil to increase the antimicrobial effectiveness at a smaller volume with the addition of electrostatic forces to attract negatively charged bacteria to positively charged EOs. Although the concept of emulsification and encapsulation sounds promising, the minimal biofilm eradication concentrations (MBECs) for the coated and uncoated emulsified products were only half a log lower than the pure isoeugenol when tested in standard laboratory medium at three temperatures (6, 12, and 25°C) and no difference was observed in carrot juice. This observation requires further exploration as the reductions in the MBEC did not correlate to observations under confocal microscopy. Of note was the morphological changes observed in the mixed biofilms of *Pseudomonas fluorescens* and *S. aureus* from uniform layers to clusters of numerous cells, which requires further research to determine if there are any implications.

As mentioned previously, the use of EOs at concentrations to exhibit sufficient antimicrobial activity has the potential to impart undesirable flavor and when applied in an FPE may also result in an excessive sensorial impact. In addition, the interactions of EOs with components of the food matrix from food debris may also impact on the applicability of EOs in food environments. Only a few studies have investigated the application of EOs to disrupt or prevent the formation of biofilms. Further research on parameters specific to industry will allow a better decision on the application of EOs as an alternative or supplementary biocontrol method.

## Concluding Remarks

While current sanitation processes are effective against planktonic cells, the potential for tolerant strains to increase due to interactions at subinhibitory levels and the potential reliance on them as antimicrobials, as the case in the health industry, is a cause for concern. The ability to eradicate established biofilms and prevent new biofilms from being formed is a challenging task which food production managers are charged with, as biofilms can present increased food safety risks. A useful tool in understanding the microbial community is metagenomics analysis of the FPE. By understanding the FPE microbiome, valuable information can be gained regarding persistence or transience of strains. This facilitates source tracking of persistent strains, can identify other microbial species that may provide either a positive or negative effect on the target strain, and can identify strains surviving the disinfection processes (Dass and Anandappa, 2017; Doyle et al., 2017). From this information, the appropriate biocontrol method can then be determined. There have been some significant advances in the development of biocontrol methods, particularly bacteriophages that have progressed to commercial products with the results of some studies validating their progression to commercialization. The use of competitive bacterial species has also showed some promising results with the concept of utilizing antagonist strains isolated from the production environment providing individualized treatment options. Bacteriocins and endolysins have also shown their ability to significantly reduce established biofilms; however, they typically require some form of purification process to achieve these results. The sensory implications of EOs at concentrations required to exert antimicrobial effects are a limiting factor in their use as a sole biocontrol method, and therefore they may find more appropriate utility as a supplementary method targeting non-food contact surfaces. However, like all biocontrol methods, efficacy can be impacted by a variety of factors including temperature or time the control method was applied for, the use of one species or multiple species biofilms, biofilm growth method, or surface matrix composition. Standardization in the assessment of novel biocontrol methods against biofilms is required, in addition to assessment under conditions reflective of FPEs before appropriate comparisons can be made.

## Author Contributions

JG and EF conceived the study and drafted the manuscript. All authors corrected and approved the manuscript.

## Conflict of Interest Statement

The authors declare that the research was conducted in the absence of any commercial or financial relationships that could be construed as a potential conflict of interest. The reviewer GP and handling Editor declared their shared affiliation.
